# Longitudinal trends in produce purchasing behavior: a descriptive study of transaction level data from loyalty card households

**DOI:** 10.1186/s12937-022-00814-9

**Published:** 2022-11-08

**Authors:** Isabel Diana Fernandez, Brent A. Johnson, Nellie Wixom, Amber Kautz, Joanne Janciuras, Steve Prevost, Jiebo Luo, Rajeev S. Ramchandran

**Affiliations:** 1 Department of Public Health Sciences, University of Rochester School of Medicine and Dentistry, Rochester, NY USA; 2 Department of Biostatistics and Computational Biology, University of Rochester School of Medicine and Dentistry, Rochester, NY USA; 3 School of Nursing, University of Rochester Medical Center, Rochester, NY USA; 4 Customer Insights, Grocery Store Collaborator, Rochester, NY USA; 5 Department of Computer Science, University of Rochester, Rochester, NY USA; 6 Department of Ophthalmology, University of Rochester School of Medicine and Dentistry, Rochester, NY USA

**Keywords:** Food purchases, Produce purchases, Loyalty card, Grocery stores, Diet, Diet monitoring, Purchasing behavior

## Abstract

**Background:**

Household food purchases (HFP) are in the pathway between the community food environment and the foods available in households for consumption. As such, HFP data have emerged as alternatives to monitor population dietary trends over-time. In this paper, we investigate the use of loyalty card datasets as unexplored sources of continuously collected HFP data to describe temporal trends in household produce purchases.

**Methods:**

We partnered with a grocery store chain to obtain a loyalty card database with grocery transactions by household from January 2016-October 2018. We included households in an urban county with complete observations for head of household age group, household income group, and family size. Data were summarized as weighted averages (95% CI) of percent produce purchased out of all foods purchased by household per month. We modeled seasonal and linear trends in the proportion of produce purchases by age group and income while accounting for repeated observations per household using generalized estimating equations.

**Results:**

There are 290,098 households in the database (88% of all county households). At baseline, the smallest and largest percent produce purchases are observed among the youngest and lowest income (12.2%, CI 11.1; 13.3) and the oldest and highest income households (19.3, CI 18.9; 19.6); respectively. The seasonal variations are consistent in all age and income groups with an April-June peak gradually descending until December. However, the average linear change in percent produce purchased per household per year varies by age and income being the steepest among the youngest households at each income level (from 1.42%, CI 0.98;1.8 to 0.69%, CI 0.42;0.95) while the oldest households experience almost no annual change.

**Conclusions:**

We explored the potential of a collaboration with a food retailer to use continuously collected loyalty card data for public health nutrition purposes. Our findings suggest a trend towards a healthier pattern in long-term food purchases and household food availability among the youngest households that may lessen the population chronic disease burden if sustained. Understanding the foods available for consumption within households allows public health advocates to develop and evaluate policies and programs promoting foods and nutrients along the life course.

**Supplementary Information:**

The online version contains supplementary material available at 10.1186/s12937-022-00814-9.

## Background

Diet-related non-communicable diseases (DRNCD) are the leading cause of morbidity and mortality globally and in the United States. Globally, 22% of all deaths and 15% of disability-adjusted life-years (DALYs) were attributed to dietary factors [1]. In the US, where DRNCD disproportionally affect older adults, racial/ethnic minorities and lower income groups, dietary factors accounted for the largest number of deaths and were the third leading cause of DALYs [2]. Of concern, dietary risk factors have remained relatively unchanged in the past 10 years [3]. Combined nutrition public health policy and programs addressing all components of the food system will contribute to the improvement of dietary intake eventually lessening the population burden of DRNCD [1, 2].

Monitoring population diet quality to identify food and nutrient inadequacies is necessary to develop and evaluate public health nutrition policies and programs. To date, self-reported dietary intake assessments, such as 24-h dietary recalls or food frequency questionnaires, are the most frequently used tools for nutrition monitoring [4]. These tools are resource intensive, prone to measurement errors and social desirability bias, and some do not capture seasonal variation of food intake [5] if assessing a few days at a time (e.g.; 24-h diet recalls, food records); making them impractical to monitor long-term dietary intake at the population level. To circumvent the limitations of self-report, the search for biomarkers of dietary intake has intensified, particularly using metabolomics [6]. Despite considerable advancement in the field, few biomarkers of specific nutrients and food groups are available and even fewer capture average long term dietary intake [7]. Together with the challenges in the collection and storage of biological samples, biomarkers of dietary intake are not yet suitable for population monitoring [8].

Food purchase data have gained popularity as an alternative method to monitor population dietary intake trends and to evaluate nutrition policies and programs [9, 10]. Food purchase data by households do not directly measure individual dietary intake but rather represents the foods available to consume by household members. People make food choices in the context of the food stream, the flow of foods from the national food supply through food processing (e.g.; manufacturers) and the community food environment (e.g.; markets, grocery stores, schools), to individual food intakes [11]. Food purchasing behavior is then considered a mediator between the community food environment, the food available to members of a household and individuals’ dietary intake [12, 13]. The mediator role of food purchasing behavior is supported by evidence of its relative concordance with individual nutrient intakes [12, 14, 15] and of providing reasonable estimates of overall diet quality when compared with self-report methods [12]. Thus, household food purchases data appears to be suitable for population monitoring and evaluation. Thus far, data on food purchases have been obtained from commercially available household food purchasing panels and have examined the quality of food purchases of nutritional assistance programs (e.g.; WIC), and the nutrient compositions of purchased foods at the brand-level, [10] associations between sociodemographic factors and household food and beverage purchases, the effect of marketing (e.g.; coupons) and public health interventions (e.g.; food taxes). A recent report, however, has identified the need to develop partnerships between nutrition researchers and independent and chain food retailers willing to share loyalty card data to support healthy eating and to advance policy and practice [16].

In this paper, we report the results of loyalty card data analyses based on a partnership between a regional supermarket chain in Western New York that accounts for 65% of total dollar food expenditure in the county (private communication with the food retailer) and an academic health center as a demonstration of these collaborations’ potential to advance public health nutrition. We chose to focus on household purchases of fresh fruits and vegetables because they are major sources of dietary fibers, antioxidants, flavonoids and polyphenols and primary components of the Healthy U.S.-, Vegetarian-, and Mediterranean-Styles Patterns recommended by the US dietary guidelines for the prevention of chronic conditions across the life course [17]. Fruit and vegetable intake is inversely associated with cardiovascular disease morbidity and mortality, and all-cause mortality [18–21]. Some studies have reported a lower incidence of pancreatic and gastric cancers, age-related macular degeneration, and type 2 diabetes [20–22] with increased fruit and vegetable consumption, although others have found weak or inconsistent associations with all cancers [18, 23] and diabetes [19]. Despite this evidence, population intake of fruits and vegetables, as reported in national nutrition surveys [24, 25], is consistently below the recommendations [17, 26]. It follows that these food groups are ideal targets of public health nutrition programs and policies for the prevention of DRNCD. The objective of this paper is to describe household fresh fruits and vegetables (hereafter produce) purchases as the interface between the community food environment and individual eating behaviors and to examine temporal and seasonal trends by demographic characteristics.

## Methods

We follow the guidelines of Strengthening the Reporting of Observational Studies in Epidemiology extension for nutritional epidemiology (STROBE-nut) [27] (Supplemental Table 1).

This is a secondary data analysis of a grocery store loyalty card database with household food transactions to describe seasonal and temporal trends in fresh produce purchases from January 2016 to October 2018 (33 months). A loyalty card program rewards frequent customers with discounts or incentives to encourage purchases at the store. The data were obtained as part of a long term collaboration between the authors and a regional grocery store chain in a Western NY county. The dataset includes all purchases of food and non-food items of loyalty cardholders by household. Purchases made by each household member with a loyalty card are merged into a unique household identifier and each household visit to a store is identified by a unique transaction number and date. Household information includes zip code, household income (nine categories: 0–14,9 K; 15–19.9 K, 20–29,9 K; 30–39,9 K; 40–49.9 K, 50–74,9 K; 75–99,9 K; 100–124.9 K; ≥ 125 K), age of head of household (six categories: 18–24.9, 25–34.9, 35- 44.9, 45–54.9, 55–74.9, 75–89), household size (1 to ≥ 6), and whether or not the household is a family (yes/no). No data on gender, education, nor race/ethnicity were available. Purchase information includes the store where the purchase was made, a short description of the item, brand name, department (e.g.; dairy), category (e.g.; yogurt), and class (e.g.; Greek yogurt). All data are de-identified except for the household 5-digit zip codes, which are updated from the National Change of Address data from the United States Postal Service. Likewise, the company updates all demographic data from households annually using commercial databases. We included households in zip codes for one mostly urban county (17 stores) and excluded all non-food related departments (e.g.; pharmacy) and households with a head of household ≥ 90 years old (1.6% of households) under the assumption that their involvement in food shopping is minimal [28] and to comply with research review board standards of using de-identified data. Additionally, we manually checked all food departments to eliminate non-food products (e.g.; candy making mold and supplies and pet foods found in bulk food department, coupons); and foods and drinks not considered for households every day consumption (produce party trays ≥ 1 pound, cheese and cold cut party trays of ≥ 2 pounds, beer kegs).

### Variables

Produce was identified as all fresh raw produce categories in the loyalty card database selected from the department ‘produce’ (excluding frozen, dried, canned, juice): summer fruit, berries, grapes, seasonal/specialty, apples and pears, salad vegetables, citrus, tropical fruit, bananas, melons, potatoes and onions, cooking vegetables, and salad leaf. To model seasonal and annual trends we estimated the weighted average of the percent produce purchases (fresh produce purchased/all foods purchased) per household across all households per month (weight = the number of foods purchased per household) for 33 months. In this way, we controlled for the effect of family size in the amount of produce purchased (larger families, more purchases). In these analyses, each unique food item purchased in a single transaction is considered to be one unit, regardless of true quantity purchased; thus, percent produce purchased represents the frequency rather than the quantity of foods purchased. Categories of age groups from the original dataset were collapsed (18–24, 25–34, 35–54, 55–74, and 75–89 years old) because exploratory analyses showed overlapping percentage produce purchases over time in the 35–44 and 45–54 age groups. A similar finding among income groups allowed for additional data reduction from nine groups to six (0–14,9 K; 15–29,9 K; 30–49,9 K; 50–74,9 K; 75–99,9 K; > 100 K).

### Statistical analyses

We describe the characteristics of loyalty card households by age of head of household, household income, family (yes/no), and household size. To assess how representative the loyalty card households were of the general population of the county, we compared the proportion of households in each household income and age of head of household groups with county data from 2017 American Community Survey (ACS) [29]. For comparison with the available ACS household income groups, we collapsed groups 15–29.9 K and 30–49.9 K into a 15–49.9 K income group. Since the ACS does not include data on age-group of head of households, we reported the county population age-groups.

We modeled temporal trends in the proportion of percent fresh produce purchases over time while accounting for repeated observations per household using generalized estimating equations. [30, 31]. The basic statistical model used to describe purchasing trends is 

**Fig. 1:**
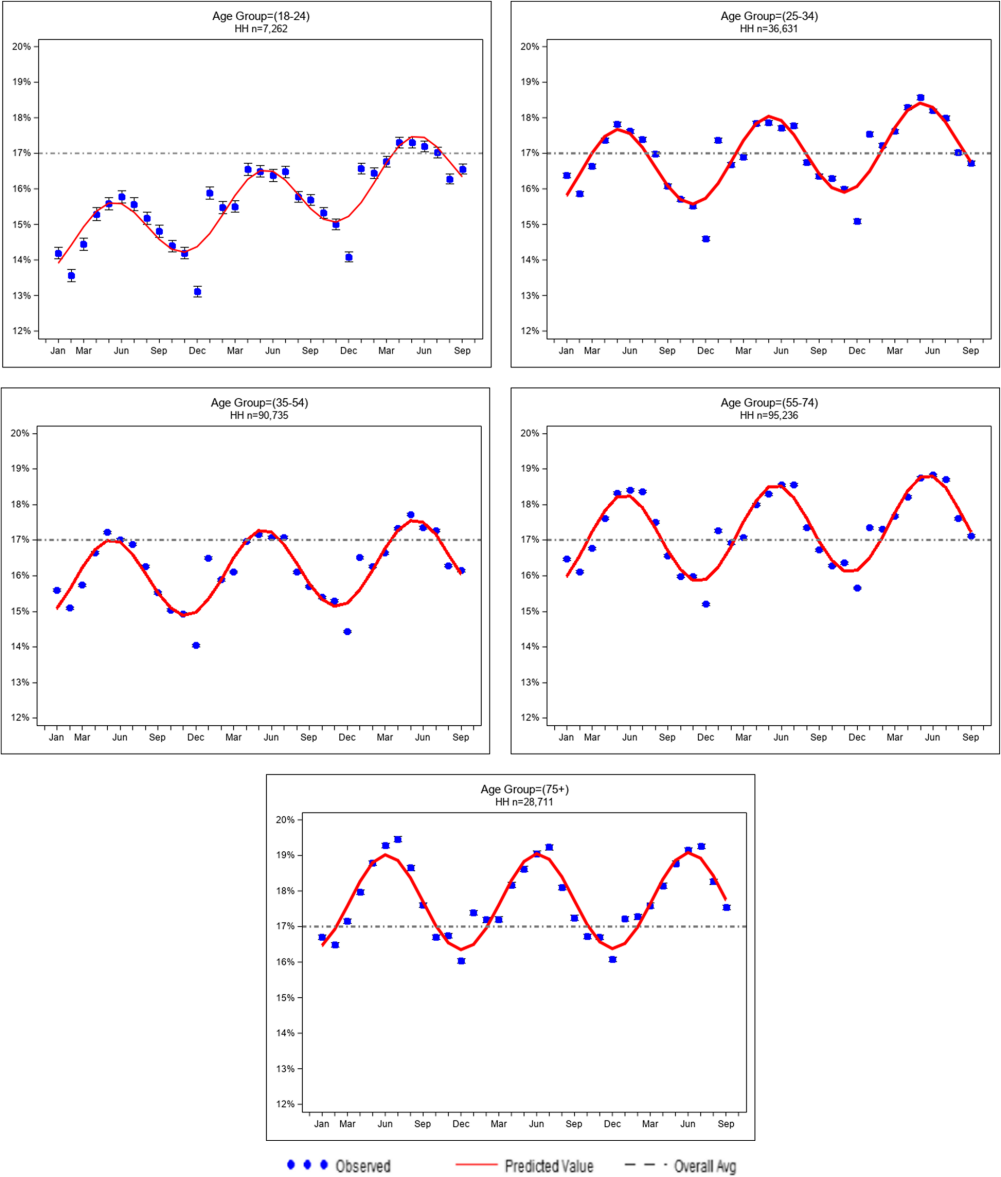
Seasonal trends in percent household produce purchased by age group (January 2016-October 2018)

**Fig. 2:**
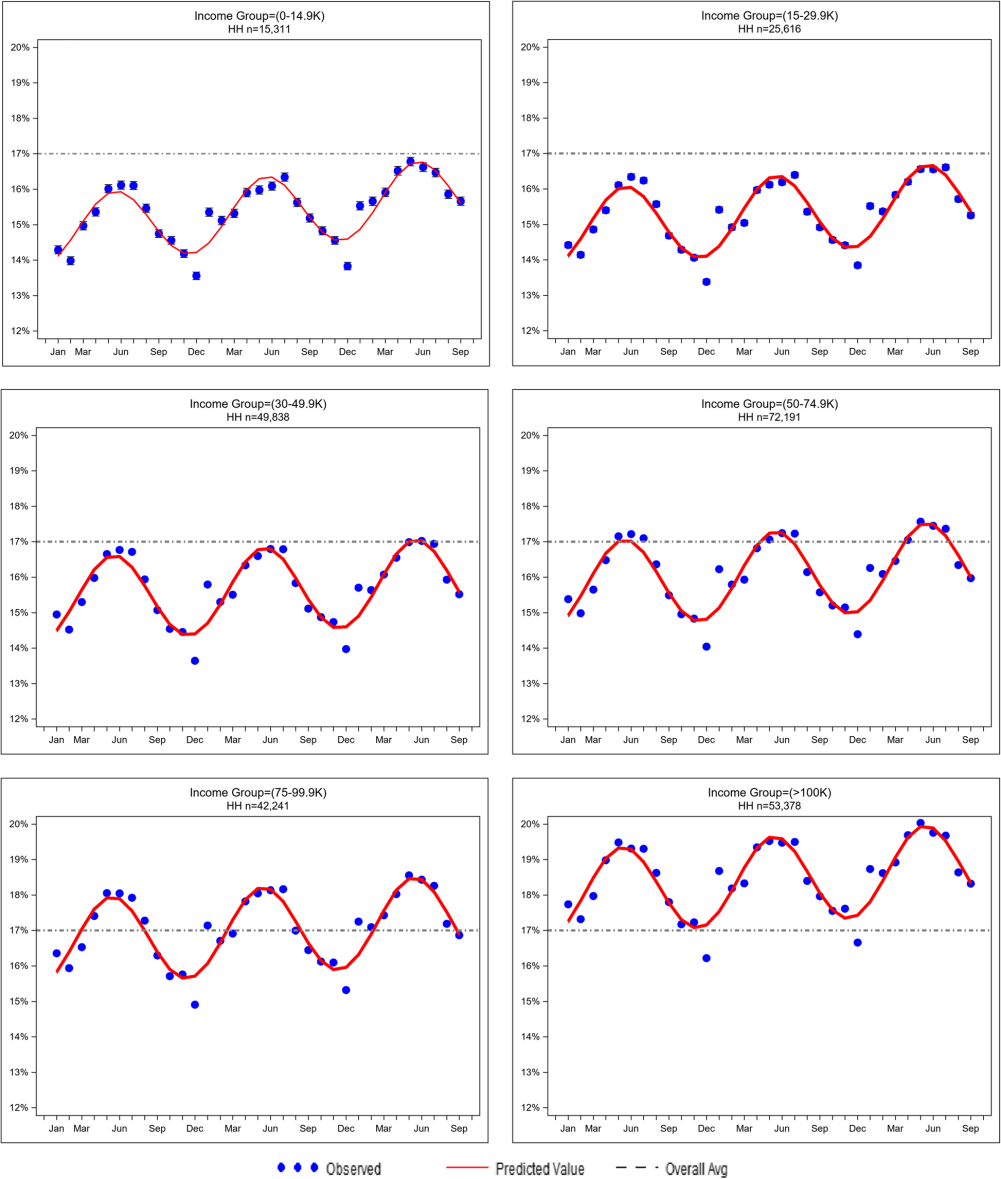
Seasonal trends in percent household produce purchased by income group (January 2016-October 2018)

Equation (1) $$\begin{array}{c}logit\left\{P\left(Y_{ij}=y_{ij}\right)\right\}\mathit\;=\mathit\;\beta_{\mathit0}+\;\beta_Ss\left(t_{ij}\right)\mathit\;+\;\beta_Lt_{ij}+\mathit\;\beta_XX_i\mathit\;+\;\beta_{XL}\left(X_{\mathit1\mathit\;}\times\;t_{ij}\right)\\\beta_Ss(t_{ij})\;=\;\beta_{s_{\mathit1}}\;\sin\;\left(\frac{2\pi t_{ij}}{12}\right)\;+\;\beta_{s_{\mathit2}}\;\cos\;\left(\frac{2\pi t_{ij}}{12}\right)\end{array}$$ 

where, ${Y}_{ij}$ is a binomial random variable representing the number of fresh produce items purchased by the *i*th household during the *j*th month based on a total of ${n}_{ij}$ food items purchased during the *j*th month. The terms in the model include time (${t}_{ij}$) recorded as an integer from 1 to 33, ${X}_{i}$ are demographic variables (age or income groups), and ${X}_{i}\times {t}_{ij}$ are covariate-by-time interactions (age group x time; income group x time; age group x income group x time). Importantly, seasonal trends of percent produce purchased $s({t}_{ij})$ are modeled as the sum of sinusoidal and cosinusoidal terms as written in the second line of Eq. (1). The unknown regression coefficients are ${\beta }_{0}, {\beta }_{{S}_{1}}, {\beta }_{{S}_{2}}, {\beta }_{L}, {\beta }_{X}, {\beta }_{XL}$ and estimated by the statistical procedure. Thus, the percent fresh produce purchasing trends are modeled as the sum of seasonal variations $({\beta }_{S})$ and linear trends (${\beta }_{L}$) over time, with the linear trends different by age and income groups. We draw statistical inference using the robust empirical sandwich covariance under a working independence correlation model. We use linear contrasts of coefficients in models via Eq. (1) that include the three-way time-by-age-by-income interaction to estimate the age-by-income slopes over the 33-month period. After rescaling the coefficients, the slopes are interpreted as the average linear change in percent produce purchased per household per year over 33 months after controlling for seasonal variation. We depicted the seasonal variations graphically by overlaying the fitted curve atop the data; in these Figs.(1 and 2), the data are summarized as weighted averages of percent produce purchased by household across all households per month and a 95% confidence interval, where weight is equal to the number of foods purchased per household. We included a horizontal line in each graph representing the grand mean of percent produce purchased by household as a reference. The analysis of repeated binomial proportions has a rich history in the statistics literature and is an appropriate tool for modeling the percent produce purchased in our sample. Because the proportion is the ratio of number of produce items purchased divided by the total number of food items purchased and re-computed on a monthly basis per household, the model controls automatically for monthly fluctuations in number of food items purchased per household. Furthermore, the precision of the proportion of produce items purchased increases as the number of total food items purchased increases. Thus, households that purchase more food items are weighted more heavily in the analysis. We analyzed data with complete observations. Models were fit in SAS statistical software (SAS Version 9.4; SAS Institute, Cary, NC). The University of Rochester Human Subjects Review Board approved all study procedures and granted a waiver of consent.

## Results

### Descriptive statistics

There are 290,098 households who are loyalty cardholders in the county, of which 4% and 9% had missing data on age of head of household and household income, respectively, and 15% had missing data on family status (yes/no) and family size. Consequently, sample size totals in Tables 1, 2 and 3 differ. Most of the head of households are in the 35–54 and 55–74 age groups (72%) while less than 3% are in the youngest age group (18-24). The largest income group among loyalty card households (28%) is 50–74.9 k, while 6% are in the lowest income bracket (0–14.9 K) (Table 1). Half of the households identified themselves as a family, 81% of them have 1–4 members (Table 2). Loyalty card households constitute 88% of all county households in 2017. The loyalty card database has a smaller proportion of households in the lower income groups (≤ $49.900) than county households, 35.5% vs 45.6%, respectively, and a larger proportion of older head of households (≥ aged 55) than the county population, 47% vs. 37%, respectively (Table 3).

**Table 1:** Characteristics of loyalty card households by age and income groups. January 2016-October 2018

	**Annual Household Income**						
**Age Head of Household** **(years)**	**0–14.9 K** % (n**)**	**15 – 29.9 K** % (n)	**30–49.9 K** % (n)	**50 – 74,9 K** % (n)	**75–99.9 K** % (n**)**	** ≥ 100 K** % (n)	**All** Row % (n)
**18–24**	6.5 (997)	4.1 (1054)	3.2 (1573)	2.2 (1559)	1.9 (804)	2.4 (1280)	2.8 (7267)
**25–34**	25.5 (3909)	19.6 (5021)	14.5 (7252)	12.0 (8709)	10.9 (4625)	13.5 (7190)	14.2 (36706)
**35–54**	33.3 (5105)	33.5 (8605)	34.8 (17346)	37.6 (27166)	34.6(14641)	33.7 (18007)	35.1 (90870)
**55–74**	23.7 (3638)	28.5 (7326)	33.4 (16667)	34.6 (26331)	43.5 (18385)	43.1 (23015)	36.8 (95362)
**75–89**	11.1 (1704)	14.3 (3675)	14.2 (7069)	11.8 (8518)	9.1 (3856)	7.4 (3950)	11.1 (28772)
**All** Column **% (n)**	5.9 (15353)	9.9 (25681)	19.3 (49907)	27.9 (72283)	16.3 (42311)	20.6 (53442)	100 (258977)

**Table 2:** Characteristics of loyalty card households by age and family characteristics

	**Family**			**Household Size**						
**Head of Household Age** **(years)**	**No** % (n)	**Yes** % (n)	**All** % (n)	**1** % (n)	**2** % (n)	**3** % (n)	**4** % (n)	**5** % (n)	** > 6** % (n)	**All** % (n)
**18–24**	1.8 (2258)	2.3 (2787)	2.1 (5045)	3.1 (1400)	1.2 (691)	1.4 (814)	2.0 (751)	2.4 (566)	3.8 (823)	2 (5045)
**25–34**	13.7 (16937)	12.4 (14990)	13.1 (31927)	22.4 (10041)	10.9 (6300)	10.3 (6045)	10.0 (3817)	11.5 (2722)	13.9 (3002)	13 (31927)
**35–54**	28.3 (34919)	42.5 (51584)	35.4 (86503)	36.8 (16467)	32.2 (18653)	35.5 (20759)	36.1 (13745)	37.6 (8897)	36.9 (7982)	35 (86503)
**55–74**	39. 7 (48919)	36.1 (43851)	37.9 (92770)	29.5 (13236)	38.6 (22369)	39.4 (23006)	41.5 (15785)	41.1 (9726)	40.0 (8648)	38 (92770)
**75–90**	16.4 (20154)	6.7 (8116)	11.6 (28270)	8.2 (3658)	17.2 (9943)	13.4 (7814)	10.3 (3927)	7.5 (1773)	5.3 (1155)	12 (28270)
**All**	50.4 (123187)	49.6 (121328)	100% (244515)	18.3 (44802)	23.7 (57956)	23.9 (58438)	15.6 (38025)	9.7 (23684)	8.8 (21610)	100% (244515)

**Table 3:** Comparison of loyalty card and county household characteristics

**Annual Household Income** ($)^*^	**Loyalty Card ** **% (n HH)**	**ACS**^a^** (2017)** [29] **% (n HH)**
0–14.9 K	6.1 (16233)	12.3 (37056)
15 – 49.9 K	29.4 (77691)	33.3 (100056)
50 – 74.9 K	27.7 (73276)	17.7 (53308)
75 – 99.9	16.2 (42785)	12.4 (37354)
≥ 100 K	20.5 (54074)	24.2 (72722)
Total HH	100 (264059)	100 (300496)
**Age-Group** (years)	**% (n HH)**	**% (n People)**^b^
18–24	3.7 (10246)	13.4 (79001)
25–34	14.7 (41048)	17.6 (103767)
35–54	34.9 (97378)	31.8 (187328)
55–74	36.0 (100248)	28.3 (166797)
75 -89	11.0 (30215)	8.9 (52183)
Total	100 (279135)	100 (589076)

^a^ *ACS* American Community Survey

^b^ ACS Does not provide data on age of head of household

^*^ Statistically significant difference based on a Chi-square test *p*-value < 0.0001 for household income group comparison between Loyalty Card and ACS

### Seasonal trends (Figs. 1 and 2)

Models for seasonal variation fit the data well for all months except for December and January, in which the model over- and under-fits the data, respectively. The seasonal trends are consistent in all age-and income-groups. A steady increase in percent produce purchased is observed from January each year reaching its peak in April through July and gradually descending until December. The differences between these seasonal peaks and valleys are between 2 and 3% produce purchased in all age and income groups. In the youngest age group (18–24 years old) the peak percent produce purchased by households is below the overall mean (17%) until the spring of 2018 (Fig. 1). In the other age groups, the peak percent produce purchased by households are above the mean for all households (17%) in the spring, particularly in the 75–89-year-old group. In the three lowest income groups, percent produce purchased are below the mean for all households regardless of season (Fig. 2). Households whose income is ≥ 100 k have a percent produce purchased always above the mean regardless of the season.

### Percent produce purchased at baseline (Supplemental Table 1)

The baseline percent produce purchased is the expected percent produce purchased by households as of January 1, 2016 (Supplemental Table 1). The youngest and oldest age groups purchased the smallest and largest percent of produce (13.5%, 95% CI 13.1–13.9; and 16.8%, 95% CI 16.6–16.9, respectively) (main effect of age group), although the trend is not consistently upward by age group. Conversely, the percent produce purchased is larger as household income increases, 13.9% (95% CI 13.6–14.1) in the lowest and 17.1% (95% CI: 17.1–17.2) is the highest household income (main effect of income group). When examining the joint contribution of age and income groups to the percent produce purchased (interaction of age and income groups), among households in age groups between 35 and 89, the higher the household income the larger the percent produce purchased. In the youngest age groups, the contribution of income to percent produce purchased does not have a consistent trend, particularly among the 18–24 year-old households in which the proportion of produce purchased by income group is the most varied. Within each household income group, although the oldest households always purchased proportionally more produce than the youngest, the effect of age on percent produce purchased does not have a monotonic trend upward. In households at each income level, those in the 25–34 year old age group purchased a larger percentage of their groceries as produce than the immediately younger (18–24) and immediately older (35–54) households. The largest difference between the highest and lowest income groups in percent produce purchased is observed among the 55–74 and 75–89 year old households (4.1% and 5.2%, respectively).

### Linear trends (Fig. 3 and Supplemental Table 1)

Over the 33 months of data, there is an upward average linear change in percent produce purchased per year of varied degrees. The youngest households increased their annual produce purchased by 0.89% (95% CI 0.74–1.04) with a steady flattening of the trend in the subsequent age groups down to the oldest households in which no change over time was observed (0.03%; 95% CI -0.02,0.08) (supplemental Table 1, main effect by age). Although the income groups experienced an annual increase in percent produce purchased, the rate of change was relatively similar across income groups, with an annual increase in produce purchased of 0.41% (95% CI 0.31–0.51) in the lowest income group to 0.29% (95% CI 0.26-0.31) in the highest one (supplemental Table 1, main effect by income). The joint contribution of age and income groups to annual average linear trend in percent produce purchased is steepest among the 18–24 year old households at each income level with more than or close to 1% a year up to the 50 K-74 K income group while the rate of annual increase slows down at higher income groups although still higher than all other age-income combinations. All other age-income combinations have average annual increase not exceeding 0.5%. Of note, the oldest households (75–89 years-old) have almost no change in the average annual percent produce purchased at all income levels.

**Fig. 3:**
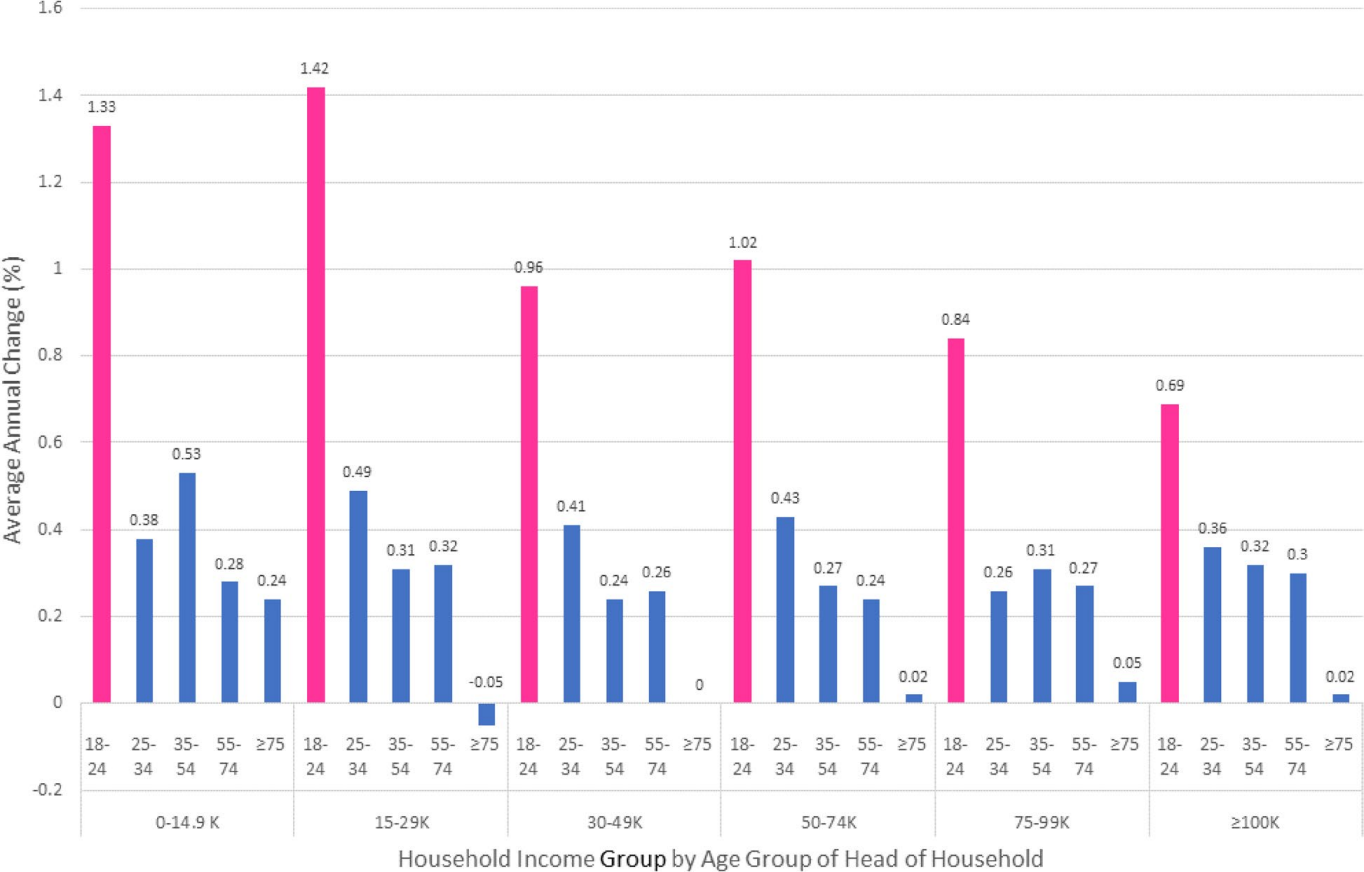
Annual rate of change in household produce purchased by income and age groups

## Discussion

We collaborated with a regional grocery store chain to explore the use of household food purchasing data from a loyalty card dataset to examine long-term population trends in food purchases. In this study, we described seasonal and linear trends in household fresh produce purchasing as a proportion of total food purchases by age of head of households and household income. We found clear seasonal trends over the 33 months of data in all age and income groups. Superimposed to seasonal variations, we observed upward annual linear trends in percent produce purchased over time in most households. The influence of household income and age of head of household was more pronounced in the overall magnitude of produce purchasing (baseline percent produce purchase as of January 2016) while change overtime seemed to be more a function of age rather than income. Older households and households with the largest income purchased a larger proportion of produce at baseline than younger and lower income households in almost all combinations but the effect of income was particularly pronounced in the two oldest household groups. The annual rate of change, however, was especially fast in the youngest households (18–25 years-old) at all levels of household income. Out of all food purchases, these young households are increasing the share of fresh produce purchased by around 1% per year while the oldest households (75–89 years-old) had an almost flat annual rate of change in produce purchasing overtime regardless of household income.

The observed seasonal trends are as expected, a peak in the harvest months with valleys in between [32]. The seasonal peaks observed are parallel to the increase in variety and volume of fruits and vegetables grown by farmers in the region who sell produce to the local groceries stores [33]. To our knowledge, no previous study described the joint effects of age of head of household and household income on linear trends in produce purchases in relation to all household food purchases over almost three years of continuously collected data. Studies present averaged data over time and show substantial methodological heterogeneity. Produce purchases have been operationalized as dollars spent as a percentage of total household food expenditures [34–37], per capita expenditures [34, 36], percent of purchased energy from produce,[38] and per capita purchased servings per 1000 purchased kilocalories [39]. Data on purchases were collected from participants’ receipts of purchases in all food outlets (grocery stores, convenient stores, restaurants, markets, etc.) for 2-4 weeks [35], self-scanned purchases in all food outlets for one year [37, 38] and participants’ diary of all food expenses for 2 weeks [34]. Despite these differences, their findings are consistent with ours in that household income consistently seems to drive produce purchasing. The wealthier the household the larger the share of produce purchased [34, 35, 40]. Income was not related to produce purchases in only one study [39] although the narrow range of household incomes in the sample may explain this finding. In studies that adjusted for race, marital status [35] and educational attainment [34, 35], the effect of income did not hold probably due to the high correlation among some of these variables. Also consistent with previous evidence is the finding that the youngest households (18–24 years old) purchase less produce than older ones but still the share of produce purchases increases with household income [37]. The 18–24 year old household’s purchasing behavior may reflect the transitional characteristics of this age group. People in this age group are more likely to be living alone for the first time at college or working lower paying jobs or both and juggling a young family; thus, favoring convenience (purchasing prepared or easy to make foods) over fresh produce [37, 41]. Another characteristic of younger shoppers is that they are value driven and tend to prioritize organic foods and pay attention to their origin and to whether food is grown sustainably, characteristics that are mostly found at natural food sources, farmers markets, and limited assortment stores [42, 43]. The later may explain why younger households with high incomes in our grocery store sample have a smaller percent produce purchased than older households with the same income. Lower income households tend to favor calorically dense foods to obtain more energy for dollar spent [44]. Regional data on barriers to buying healthy food support our interpretation. A little over 50% of area residents with income under $25 K report cost as the most important barrier compared to 20% with an income over $75,000. A higher proportion of area residents aged 18–24 years old, relative to those aged 64 years and older, reported cost (50% vs. 18%) and limited time to shop and prepare meals (30% vs. 5%) as barriers to purchasing healthier foods [33].

The current study brings to light the effect of household age and income on long-term longitudinal trends in produce purchasing behavior. We found that the rate of change over time seems more a function of age rather than income. The fastest rate of growth among the youngest households (18–25 year-olds) across income levels seems to indicate households’ decisions to purchase produce among this age group are influenced by factors other than income (e.g.; adaptations over time, more education over the 33 months period). We do not have data to evaluate whether this upward trend is the cross-sectional effect of age or a cohort effect. If the latter, the findings among 18–24 year-old households highlights a healthy trend of increased fresh produce purchases in the future. At an average rate of 1% increase per year in the proportion of fresh produce purchased out of all other food products, in 10 years the share would increase by 10% of all food purchases. Assuming that household produce purchases represent produce availability in homes to consume, the upward trend in household produce purchases suggests a trend towards a healthier pattern in long term food consumption at the population level that may contribute to a lesser chronic disease burden as the 18–24 year-old head of household cohort ages. In all other age and income groups, the upward change is similar and relatively slow especially in the 75–89 year-old household. The oldest households do not change their purchasing behavior over time and income does not factor in, perhaps reflecting a generalized resistance to change among older adults [45].

Contrary to previous literature [34–38], our food purchase data were obtained from a single grocery store chain in the county, thus missing purchases from other food outlets. We expect the peaks in the harvest months observed might be underestimated because produce bought at farmers markets or through community supported agriculture shares are not included. We do not anticipate, however, other effects of missing food outlets to be substantial given the store’s large contribution to total dollar food expenditure in the county, and the broad representation of the county’s households (88%) by loyalty card members. Additionally, loyalty card households visit the stores a median of 5.1 times per month while 75% do so more than 2.4 times (data not shown) potentially indicating that households have the opportunity to purchase perishable items such as fresh produce in this single grocery store chain without relying on other sources of fresh produce. Nevertheless, the lack of information on foods purchased at other food outlets can have a number of different implications for our study and for the potential use of loyalty card databases for population surveillance. In the best case scenario, loyalty card households who regularly shop at multiple grocery stores (e.g.; price shopping) would purchase the same type of foods and, consequently, the purchased proportions would not change and the ranking of household percent produce purchased based only on the loyalty card database would be an acceptable representation of households food purchasing experience in the county. Conversely, loyalty card households can selectively purchase different type of foods at different outlets, in which case the percent produce purchased based on the loyalty card database would not capture the true county food purchasing behavior. Also contrary to large consumer panel studies with a representative sample of the population [34, 37, 38, 41], our data has a smaller representation of lower income households. Future studies should collect receipts of all food purchases in a sample cohort of households with and without loyalty card membership to elucidate whether household food purchasing rankings differ from those obtained only from the grocery store chain. Finally, missing timepoints, a month with missing percent produce purchased because a household did not purchase food at the store, would have an effect on the study results if the missing food items in a given month were associated with observed and/or unobserved household characteristics. Although it is not clear how and why this would be true, it is important to keep it in mind in the interpretation of the results of an observational study. A meaningful methodological advantage of the loyalty card datasets is the absence of reporting errors. Datasets obtaining purchase information from food receipts or scanners provided by study participants are prone to random omissions or social desirability bias. In addition, the grocery store dataset has all household food purchases continuously collected for 33 months; thus, capturing usual long term shopping behaviors.

## Conclusions

Our study demonstrates the feasibility of a collaboration between researchers and a food retailer to use loyalty card data for public health nutrition purposes. Since household food purchases are in the intermediate pathway between the community food environment and household food availability for consumption, household food purchases from grocery store databases have the potential to be a more unbiased and representative estimate of long-term population dietary patterns and be more sensitive to programs and policies than periodic surveys of self-reported dietary intake. In larger markets, collaboration with all major food outlets would be necessary to assess population purchasing trends. Household purchasing data from grocery store databases offer many possibilities for public health nutrition research. For example, purchasing trends of all other food groups may be examined to estimate the overall quality of foods available for consumption within households, proportion of food dollars allocated to each food group, differences by additional demographic characteristics, the effect of price fluctuations in foods purchases, etc. Through the application of data science methods, grocery store foods, including ready-to-eat and unprepared, can be linked with USDA nutrient composition databases for a variety of uses related to better understanding the linkage between foods available for consumption and the health status of a population. Understanding the food available within households for consumption allows public health advocates to emphasize and promote the purchasing of foods containing critical nutrients for specific stages along the life course such as promoting the purchase of foods rich in lutein and zeaxanthin for the secondary prevention of age-related macular degeneration or rich in folate among women of reproductive age for the primary prevention of birth defects.

## Supplementary Information

**Additional file 1: Table 1.** STROBE-nut: An extension of the STROBE statement for nutritional epidemiology.

**Additional file 2: Supplemental Table 1**. Baseline^1^ and Rate of Change^2^ in Percent Produce Purchased by Age and Income Groups.

## Data Availability

The data analyzed in the current study are not publicly available because these data are proprietary. Data are however available from the authors upon reasonable request and with permission of the grocery store leadership.

## Abbreviations

DRNCD: Diet-related non-communicable diseases
US: United States of America
DALYs: Disability-Adjusted Life-Years
ACS: American Community Survey

## References

[1] Global Burden of Disease 2017 Diet Collaborators (2019). Health effects of dietary risks in 195 countries, 1990–2017: a systematic analysis for the Global Burden of Disease Study 2017. Lancet.

[2] Mokdad AH, Ballestros K, Echko M, Glenn S, Olsen HE, Mullany E (2018). The State of US Health, 1990–2016: Burden of Diseases, Injuries, and Risk Factors Among US States. JAMA.

[3] Global Burden of Disease 2019 Risk Factors Collaborators (2020). Global burden of 87 risk factors in 204 countries and territories, 1990–2019: a systematic analysis for the Global Burden of Disease Study 2019. Lancet..

[4] Dao MC, Subar AF, Warthon-Medina M, Cade JE, Burrows T, Golley RK (2019). Dietary assessment toolkits: an overview. Public Health Nutr.

[5] National Cancer Institute. Epidemiology and Cancer Research Program. Dietary Assessment Primer. Available from: https://dietassessmentprimer.cancer.gov/learn/season.html.

[6] Brennan L (2018). Moving toward Objective Biomarkers of Dietary Intake. J Nutr.

[7] Prentice RL (2020). Dietary Assessment and Opportunities to Enhance Nutritional Epidemiology Evidence. Ann Intern Med.

[8] Pfeiffer CM, Lacher DA, Schleicher RL, Johnson CL, Yetley EA (2017). Challenges and Lessons Learned in Generating and Interpreting NHANES Nutritional Biomarker Data. Adv Nutr.

[9] Economic Research Service (ERS), U.S. Department of Agriculture (USDA). 2012–2013 National Household Food Acquisition and Purchase Survey (FoodAPS) [updated August 20, 2019]. Available from: https://www.ers.usda.gov/foodaps.

[10] Bandy L, Adhikari V, Jebb S, Rayner M (2019). The use of commercial food purchase data for public health nutrition research: a systematic review. PLoS One.

[11] National Cancer Institute. The Healthy Eating Index – What is the Food Stream? https://epi.grants.cancer.gov/hei/uses.html#stream. Updated July 24, 2020. Accessed 10 Aug 2020.

[12] Appelhans BM, French SA, Tangney CC, Powell LM, Wang Y (2017). To what extent do food purchases reflect shoppers' diet quality and nutrient intake?. Int J Behav Nutr Phys Act.

[13] Volpe R, Okrent A. Assessing the Healthfulness of Consumers’ Grocery Purchases. USDA, Economic Research Service 2012, EIB-102. Available online: https://ageconsearch.umn.edu/record/262129/. (Accessed 20 June 2021).

[14] Eyles H, Jiang Y, Ni MC (2010). Use of household supermarket sales data to estimate nutrient intakes: a comparison with repeat 24-hour dietary recalls. J Am Diet Assoc.

[15] Ransley JK, Donnelly JK, Khara TN, Botham H, Arnot H, Greenwood DC (2001). The use of supermarket till receipts to determine the fat and energy intake in a UK population. Public Health Nutr.

[16] Hecht A, Moran A, Snyder E, Lot tM, Arm K, Story M, et al. National Research Agenda to Support Healthy Eating through Retail Strategies. Durham, NC: Healthy Eating Research. Available at http://healthyeatingresearch.org. 2020. 10.3390/ijerph17218141 PMC7663573 33158134

[17] U.S. Department of Agriculture and U.S. Department of Health and Human Services. Dietary Guidelines for Americans, 2020-2025. 9th Edition. December 2020. Available at DietaryGuidelines.gov.

[18] Aune D, Giovannucci E, Boffetta P, Fadnes LT, Keum N, Norat T (2017). Fruit and vegetable intake and the risk of cardiovascular disease, total cancer and all-cause mortality-a systematic review and dose-response meta-analysis of prospective studies. Int J Epidemiol.

[19] Martinez-Gonzalez MA, de la Fuente-Arrillaga C, Lopez-Del-Burgo C, Vazquez-Ruiz Z, Benito S, Ruiz-Canela M (2011). Low consumption of fruit and vegetables and risk of chronic disease: a review of the epidemiological evidence and temporal trends among Spanish graduates. Public Health Nutr.

[20] Veronese N, Solmi M, Caruso MG, Giannelli G, Osella AR, Evangelou E (2018). Dietary fiber and health outcomes: an umbrella review of systematic reviews and meta-analyses. Am J Clin Nutr.

[21] Partula V, Deschasaux M, Druesne-Pecollo N, Latino-Martel P, Desmetz E, Chazelas E (2020). Associations between consumption of dietary fibers and the risk of cardiovascular diseases, cancers, type 2 diabetes, and mortality in the prospective NutriNet-Sante cohort. Am J Clin Nutr..

[22] Cho E, Seddon JM, Rosner B, Willett WC, Hankinson SE (2004). Prospective study of intake of fruits, vegetables, vitamins, and carotenoids and risk of age-related maculopathy. Arch Ophthalmol.

[23] Wang X, Ouyang Y, Liu J, Zhu M, Zhao G, Bao W (2014). Fruit and vegetable consumption and mortality from all causes, cardiovascular disease, and cancer: systematic review and dose-response meta-analysis of prospective cohort studies. BMJ.

[24] Bowman SA, Clemens JC, Friday JE, Schroeder N, Shimizu M, LaComb RP, Moshfegh AJ. Food Patterns Equivalents Intakes by Americans: What We Eat in America, NHANES 2003–2004 and 2015- 2016. Food Surveys Research Group Dietary Data Brief No 20. 2018.

[25] Lee-Kwan SHML, Blanck HM, Harris DM, Galuska D (2017). Disparities in State-Specific Adult Fruit and Vegetable Consumption-United States, 2015. Morb Mortal Wkly Rep.

[26] Dietary Guidelines Advisory Committee. Scientific Report of the 2020 Dietary Guidelines Advisory Committee: Advisory Report to the Secretary of Agriculture and the Secretary of Health and Human Services. Washington: U.S. Department of Agriculture, Agricultural Research Service; 2020.

[27] Lachat C, Hawwash D, Ocké MC, Berg C, Forsum E, Hörnell A (2016). Strengthening the Reporting of Observational Studies in Epidemiology - nutritional epidemiology (STROBE-nut): An extension of the STROBE statement. Nutr Bull.

[28] Granic A, Mendonca N, Hill TR, Jagger C, Stevenson EJ, Mathers JC (2018). Nutrition in the Very Old. Nutrients.

[29] U.S. Census Bureau. American Community Survey. Selected Economic Characteristics. https://data.census.gov/cedsci/table?d=ACS%205-Year%20Estimates%20Data%20Profiles&table=DP05&tid=ACSDP5Y2017.DP03&g=0400000US36_0500000US36055&vintage=2017. 2017.

[30] Liang KY, Zeger SL (1986). Longitudinal Data-Analysis Using Generalized Linear-Models. Biometrika.

[31] Zeger SL, Liang KY (1986). Longitudinal data analysis for discrete and continuous outcomes. Biometrics.

[32] Valpiani N, Wilde P, Rogers B, Stewart H (2015). Patterns of fruit and vegetable availability and price competitiveness across four seasons are different in local food outlets and supermarkets. Public Health Nutr.

[33] Penisse M. Food and Health Connection. Final Report. Common Ground Health. 2019.

[34] Blisard N, Hayden S, Jolliffe D. Low-Income Households Expenditures on Fruits and Vegetables. USDA. Economic Research Service. Agricultural Economic Report Number 833. 2004.

[35] French SA, Tangney CC, Crane MM, Wang Y, Appelhans BM (2019). Nutrition quality of food purchases varies by household income: the SHoPPER study. BMC Public Health.

[36] French SA, Wall M, Mitchell NR (2010). Household income differences in food sources and food items purchased. Int J Behav Nutr Phys Act.

[37] Kuhns A, Saksena M. Food Purchase Decisions of Millennial Households Compared to Other Generations, EIB-186, U.S. Department of Agriculture, Economic Research Service. 2017.

[38] Pechey R, Monsivais P (2015). Supermarket Choice, Shopping Behavior, Socioeconomic Status, and Food Purchases. Am J Prev Med.

[39] Rankin J, Winnet R, Anderson E (1998). Food purchase patterns at the supermarket and their relationship to family characteristics. J Nutr Educ.

[40] Pechey R, Monsivais P, Ng YL, Marteau TM (2015). Why don't poor men eat fruit? Socioeconomic differences in motivations for fruit consumption. Appetite.

[41] Cook R (2011). Tracking demographics and US fruit and vegetable consumption patterns.

[42] Mintel Report (2019). Perimeter of the Store.

[43] Fernandez C. Supermarkets and Grocery Stores in the US. US Industry (NAICS) Report 44511. IBISWorld Inc. 2020.

[44] Grummon AH, Taillie LS (2017). Nutritional profile of Supplemental Nutrition Assistance Program household food and beverage purchases. Am J Clin Nutr.

[45] McCrae RR, Costa PT, Ostendorf F, Angleitner A, Hrebickova M, Avia MD (2000). Nature over nurture: temperament, personality, and life span development. J Pers Soc Psychol.

