# Supplementary material for: Longitudinal trends in produce purchasing behavior: a descriptive study of transaction level data from loyalty card households
Source: Nutr J. 2022 Nov 8;21:67. doi: 10.1186/s12937-022-00814-9 (PMC9644457; doi:10.1186/s12937-022-00814-9)
Supplement: Supplementary file 2 — Additional file 2: Supplemental Table 1. Baseline1 and Rate of Change2 in Percent Produce Purchased by Age and Income Groups. [file 12937_2022_814_MOESM2_ESM.docx]

| Supplemental Table 1. Baseline^1^ and Rate of Change^2^ in Percent Produce Purchased by Age and Income Groups. | | | | | | | | | | | | | | |
| --- | --- | --- | --- | --- | --- | --- | --- | --- | --- | --- | --- | --- | --- | --- |
|  | **Interaction by Age and Income Groups**  % (95% CI) | | | | | | | | | | | | **Main effect by Age** | |
| **Household Income Groups** | **0-14.9 K** | | **15-29 K** | | **30-49 K** | | **50-74 K** | | **75-99 K** | | **≥100 K** | |  |  |
| **Age of Head of Household** | Baseline | Annual Change | Baseline | Annual Change | Baseline | Annual Change | Baseline | Annual Change | Baseline | Annual Change | Baseline | Annual Change | Baseline | Annual Change |
| **18-24 years old** | 12.2  (11.1-13.3) | 1.36  (0.78,1.93) | 11.3  (10.4-12.2) | 1.42  (0.98,1.86) | 13.4  (12.5-14.2) | 0.94  (0.56,1.31) | 12.7  (11.9-13.4) | 0.99  (0.68,1.30) | 13.6  (12.5-14.6) | 0.84  (0.44,1.25) | 15.0  (14.2-15.8) | 0.69  (0.42,0.95) | 13.5  (13.1-13.9) | 0.89  (0.74,1.04) |
| **25-34 years old** | 15.1  (14.5-15.6) | 0.31  (0.07,0.56) | 14.7  (14.3-15.1) | 0.44  (0.27,0.61) | 14.9  (14.6-15.2) | 0.37  (0.25,0.50) | 14.9  (14.7-15.1) | 0.40  (0.30,0.50) | 15.8  (15.5-16.1) | 0.23  (0.09,0.36) | 16.7  (16.5-17.0) | 0.34  (0.24,0.44) | 15.5  (15.4-15.6) | 0.35  (0.30,0.40) |
| **35-54 years old** | 13.4  (13.0-13.9) | 0.50  (0.33,0.68) | 13.7  (13.4-13.9) | 0.28  (0.18,0.39) | 14.1  (14.0-14.3) | 0.20  (0.14,0.26) | 14.5  (14.4-14.6) | 0.24  (0.20,0.28) | 15.1  (15.0-15.3) | 0.29  (0.24,0.34) | 16.2  (16.1-16.3) | 0.30  (0.26,0.35) | 15.0  (14.9-15.0) | 0.27  (0.25,0.29) |
| **55-74 years old** | 13.7  (13.2-14.1) | 0.23  (0.05,0.40) | 13.8  (13.5-14.1) | 0.30  (0.19,0.40) | 14.4  (14.2-14.6) | 0.23  (0.17,0.29) | 15.1  (15.0-15.3) | 0.22  (0.18,0.26) | 16.2  (16.1-16.3) | 0.27  (0.22,0.32) | 17.8  (17.7-18.0) | 0.32  (0.28,0.36) | 16.1  (16.0-16.1) | 0.27  (0.25,0.29) |
| **75-89 years old** | 14.1  (13.5-14.8) | 0.17  (-0.07,0.41) | 15.0  (14.6-15.4) | -0.09  (-0.23,0.05) | 15.8  (15.5-16.1) | -0.02  (-0.12,0.07) | 16.7  (16.5-17.0) | 0.01  (-0.07,0.10) | 17.3  (16.9-17.7) | 0.07  (-0.05,0.18) | 19.3  (18.9-19.6) | 0.06  (-0.06,0.18) | 16.8  (16.6-16.9) | 0.03  (-0.02,0.08) |
| **Main effect by Income** | 13.9  (13.6-14.1) | 0.41  (0.31,0.51) | 14.0  (13.9-14.2) | 0.29  (0.23,0.36) | 14.5  (14.4-14.6) | 0.21  (0.18,0.25) | 15.0  (14.9-15.0) | 0.22  (0.20,0.25) | 15.8  (15.7-15.9) | 0.26  (0.23,0.29) | 17.1  (17.1-17.2) | 0.29  (0.26,0.31) |  |  |
| \| ^1^Expected percent produce purchased on January 2016 adjusted for temporal trends. ^2^Expected linear change in percent fresh produce purchased per year over 33 months adjusted for seasonal trends. \| \| --- \| | | | | | | | | | | | | | | |
|  | | | | | | | | | | | | | | |
